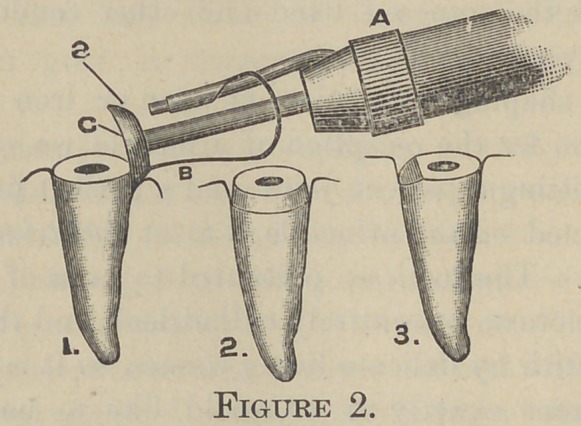# Collar Crowns and Preparation of Roots

**Published:** 1893-04

**Authors:** C. W. Jones

**Affiliations:** St. Paul, Minn.


### Collar Crowns and Preparation of Roots.

BY C. W. JONES, D.D.S., ST. PAUL, MINN.

Read before the Minnesota State Dental Association, July, 1892.

The attachment of artificial crowns to natural roots has probably attracted more attention from the dental profession the past few years than any other subject known to dental art. And in speaking of crowns I mean to include bridge work, for bridge work is nothing more nor less than the addition of one or more crowns, or dummies as they are called, to another crown or series of crowns adjusted to the natural roots; and in my mind requires as little or less mechanical ingenuity for one who has mastered a single crown than does the simplest piece in dental prosthesis.

Dentists have made great progress, and I may say have come very near perfection in the methods employed and results obtained in the construction of crowns so far as is required for appearance, firmness and permanency; but looking at it from a hygienic point of view there is yet room for great improvement, and any of you will be convinced of this fact by looking at the turgid condition of the gums where a crown has been worn for a few years, or even in many cases a few weeks. This is especially true with a great majority of collar crowns. I think I would not be far from right in saying that ninety per cent. of gold crowns or porcelain crowns with collar which are a success so far as appearance and being firmly and permanently fastened to the root is concerned, are a failure with respect to leaving the natural parts, in which they come in close contact, in a normal and healthful condition. No one will question the superiority of this kind of crown in point of strength as well as protection to the root from further decay. Yet I hear repeatedly from dentists of high authority the prediction that these crowns will not be put on so universally in the future as they have been in the past, for the reason of this inflammation produced at the gingival margin of the gum from the collar. A condition which finally results in absorption of process, pyorrhœa alveolaris, loosening of the teeth and their ultimate destruction.

"At the annual meeting of the American Dental Association at Saratoga last summer, Dr. E. Parmly Brown related a case where a lady came to his office and asked him to excise and crown a lateral incisor for her on account of a bad discoloration resulting from a large gold filling. The same lady had previously visited several New York dentists, all of whom refused to cut off the crown, knowing that the consequences of putting a band on the root would be worse than to have the gum in a healthy condition, even with a bad looking filling. Dr. Brown, after carefully considering the case, devised a scheme by which he fitted a band of platinum almost infinitesimally thin, then burnishing close to the sides of the root and entirely under the gum in front banked on a porcelain tooth. By constructing a crown in this way Dr. Brown considered himself justified in performing the operation, and did so, much to the gratification of his patient."

It is very strange that so little has been said or done on this subject of properly preparing roots for the reception of crowns and a fine adjustment of the band at the gum margin with a view to keeping the gum and contiguous parts in a normal and healthful condition. Nothing in dentistry demands finer manipulation. If we take a practical consideration of the subject we will see that our knowledge of anatomy, histology and pathology, as well as mechanical and artistic skill will be called into play and is necessary for a correct treatment of the case in hand and a proper performance of the operation.

Listen to what is said on this subject by some authors. In August *Cosmos*, 1891: "When the root has been properly shaped by trimming off overhanging points, encircle the root with a stout band, etc." Then whole pages are devoted to different kinds of crowns and methods of constructing them. Evans' "Crown and Bridge Work" has only two pages devoted to the preparation of roots for crowning, and in these two pages is given a description of root-trimmers which are almost entirely useless. In nearly all instances where illustrations are used the cuts represent crowns in position showing perfect adjustment and finish at the edge of the band and root. This is as it should be, but it is not practical or possible by present methods.

The most frequent causes leading to failure of collar crowns may be enumerated as follows:

1. Imperfectly prepared roots.
2. Low carat gold for band.
3. Roughly finished or porous band.
4. Band impinging on peridental membrane.
5. Failure to follow gum margin evenly.
6. Faulty articulation.
7. Failure to restore anatomical contour.

If we bear in mind the anatomy of a tooth we see that the exposed portion of dentine that projects above the gum is entirely covered by a shell of enamel, which is also carried a little below the margin of the gum and is there overlapped by cementum. Now in restoring a broken down crown we cannot do better than imitate nature as closely as possible. The enamel should be entirely removed under the gum, leaving the cement uninjured and allowing our gold band to run up under the gum and the sharp edge overlapped by the cement, as was the case with the natural enamel. In that case we would have no irritation whatever, provided our gold is twenty-three carat fine and smoothly polished.

In many cases it will be necessary to not only remove all the enamel, but also cut into the dentine, leaving a shoulder, then a band of considerable thickness can be used, twenty-six gauge or even thicker, thus giving additional strength and rendering a more perfect contour possible. In no case should the sharp edge of the band project toward or come in contact with the peridental membrane, but should fall inside of the line of cement as did the natural enamel. But the smooth surface of the band about one-eighth of an inch from the edge at the point where it leaves the gum margin can be brought to bear considerably against the gum without danger of irritation, and would be beneficial by keeping collections from finding their way up under the free margin of the gum.

It is an important point to preserve the anatomical contour of the crown. We will be aided materially in this by cutting and soldering the band to the shape of a truncated cone, fitting the narrowest portion to the neck of the tooth. In a late number of the *Dental Review* Dr. Black has an essay on "The Proximate Spaces and Gum Septum." The points brought out by Dr. Black are equally applicable to crowns as well as to the contour of fillings. The crown should be so shaped at the proximate spaces as to allow the gum septum to retain its natural position, and should come in close contact with the adjoining tooth near the grinding surface so as to prevent any food from being wedged down at that point and produce irritation.

In preparing roots the gum should be left in as healthy and normal a condition as possible. If in reducing the root it is necessary to remove a portion of the peridental membrane, the injury will be of a traumatic nature, and will, in all probability, heal by first intention and no grave results are to be anticipated. But the laceration of the gum margin by being ground or chopped up by corundum wheels and the like is an injury of considerable seriousness, the healing of which brings about an extensive cicatrix which contracts and is absorbed on the slightest provocation. Then we have the exposed band and other conditions which I have previously mentioned.

If we were shaping a soft piece of wood or iron perfectly cylindrical in form for the reception of a ferrule we would have no difficulty in getting a perfect joint and a perfect fit with instruments constructed on the principle of most *root trimmers* that are on the market. The tooth as presented to us is of a very dense and varied structure, not entirely cylindrical, and the instrument is interefered with by delicate living tissues, so it is very difficult to prepare a root exactly as we would like to have it for the reception of the band. For this purpose I have devised an instrument illustrated in fig. 1 and fig. 2. The cutting tool C, is concavo-convex in shape, with its inner face cut after the manner of a rasp-file, and tempered to the hardest steel temper. This in rapid rotation attacks the enamel rods on their ends and easily loosens them along their line of cleavage, the portion above the gum having been previously reduced by corundum wheels. The head is easily detached from the mandrel by unscrewing, to be replaced by another of different size or to be used on a mandrel in the right-angle handpiece. The shield S, is interposed between the cutting tool and gum, and is held by the flexible or spring

FIGURE 1.

arm B. It closely embraces the cutting tool, and passes forward of its cutting face, but on slight pressure of the instrument against the root the shield passes back, allowing the cutting tool to trim high under the gum. A is a ferrule with an elongation for attaching the shield, and fits over the nose of the handpiece. I have three sizes—one for bicuspids and laterals, one for incisors and cuspids and one for molars.

By this principle no attempt is made to remove the shoulder on all sides of the root at once, but one side is reduced at a time so that the original contour of the root can be preserved, or it may be changed to any desirable outline as in 2, of fig. 2, at the same time leaving the delicate mucous membrane—which covers the gum and is contiguous with the peridental membrane—intact.

FIGURE 2.

Having trimmed a root in this way, a collar crown or ready-made crown, such as the Rynear or Evans seamless crown, can be readily adapted, leaving a smooth and unbroken line of union between root and crown, while the pain usually attending this operation is reduced to the minimum. A root may also be readily faced above the gum margin as in 3, of fig. 2, for the reception of a Logan or Ludwig crown, leaving the joint between porcelain and root above the gum margin and out of sight.

## Figures and Tables

**Figure 1. f1:**
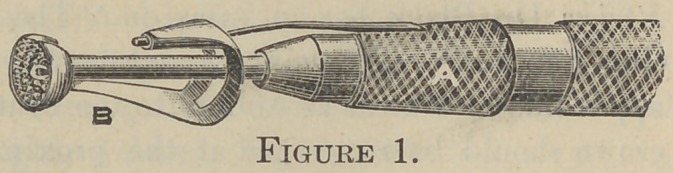


**Figure 2. f2:**